# Detection of pan-azole resistant *Aspergillus fumigatus* in horticulture and a composting facility in Belgium

**DOI:** 10.1093/mmy/myae055

**Published:** 2024-05-20

**Authors:** Hanne Debergh, Philippe Castelain, Karine Goens, Paulien Lefevere, Jessie Claessens, Elien De Vits, Marc Vissers, Liesbet Blindeman, Charlotte Bataille, Claude Saegerman, Ann Packeu

**Affiliations:** Mycology and Aerobiology, Sciensano, 1050 Brussels, Belgium; Fundamental and Applied Research for Animal and Health (FARAH) Center, University of Liège, 4000 Liège, Belgium; Unit Risk & Health Impact Assessment, Sciensano, 1050 Brussels, Belgium; Mycology and Aerobiology, Sciensano, 1050 Brussels, Belgium; Mycology and Aerobiology, Sciensano, 1050 Brussels, Belgium; Mycology and Aerobiology, Sciensano, 1050 Brussels, Belgium; Mycology and Aerobiology, Sciensano, 1050 Brussels, Belgium; Ornamental Plant Research, PCS, 9070 Destelbergen, Belgium; Ornamental Plant Research, PCS, 9070 Destelbergen, Belgium; CRA-W, Plant and Forest Health Unit, 5030 Gembloux, Belgium; Fundamental and Applied Research for Animal and Health (FARAH) Center, University of Liège, 4000 Liège, Belgium; Mycology and Aerobiology, Sciensano, 1050 Brussels, Belgium; BCCM/IHEM, Mycology and Aerobiology, Sciensano, 1050 Brussels, Belgium

**Keywords:** *Aspergillus fumigatus*, azole resistance, horticulture, agriculture, compost

## Abstract

Azole resistance in *Aspergillus fumigatus* (ARAf) is becoming a worldwide health threat due to increasing occurrence in the environment. However, environmental surveillance programs are not commonly in place and are lacking in Belgium. Since no data on the occurrence of ARAf and the presence of hotspots for the selection of azole resistance is available in Belgium, a first study on the prevalence of ARAf in the environment was conducted. A total of 232 air and compost or soil samples were taken from two composting facilities, and from horticultural and agricultural crops. The azole susceptibility pattern was determined using the EUCAST method (E. Def. 9.4), and the c*yp*51A gene and its promotor region were sequenced in *A. fumigatus* isolates with phenotypic azole resistance. Six pan-azole-resistant *A. fumigatus* isolates were identified, originating from compost and horticultural crops. Four isolates carried the TR34/L98H mutation, and one isolate carried the TR46/Y121F/T289A mutation. However, we did not observe any ARAf isolates from agricultural crops. In conclusion, this study reported the first TR34/L98H and TR46/Y121F/T289A mutation isolated from a composting facility and horticulture in Belgium. The implementation of standardization in environmental surveillance of *A. fumigatus* on a European level would be beneficial in order to identify hotspots.

## Introduction

*Aspergillus fumigatus* is a widespread saprophytic mould that naturally lives on decaying plant material and in soil.^1^ This highly sporulating mould is commonly found in compost from household and green waste, mouldy hay, and woodchips.^2,3^ Due to its thermotolerance, *A. fumigatus* is able to proliferate at high temperatures up to 60°C during the composting process.^4^ It is widespread in the air microflora and can also be isolated from water and soil.^5–9^ This opportunistic human pathogen can cause a variety of diseases in both immunocompromised and immunocompetent patients, from allergic conditions to acute and chronic angio-invasive pulmonary aspergillosis (IA) or chronic bronchopulmonary aspergillosis with high morbidity and mortality.^10,11^ Studies have shown that azole resistance in both clinical and environmental isolates has increased since the late 1990s in Europe and is currently considered as a growing global health threat due to lack of treatment options.^2,11–16^ Like any other antibiotic resistance, the development of azole-resistant *A. fumigatus* (ARAf) can occur via the patient route, which takes place when resistance develops through prolonged azole treatment. In 2009, Snelders et al. introduced the possible environmental origin of azole resistance in *A. fumigatus* due to the use of environmental azole fungicides.^9^ Since then, it has been demonstrated that the majority of cases of azole-resistant disease arise when an azole-naive patient is infected with an azole-resistant strain from the environment, which is frequently found in air and soil.^8–10,16–18^

Nowadays, the widespread use of azole fungicides in the environment is considered one of the cornerstones in the development of azole resistance in *A. fumigatus*.

In agriculture, azole fungicides, such as tebuconazole (107534-96-3), epoxiconazole (133855-98-8), propiconazole (60207-90-1), and bromuconazole (116255-48-2), are or have been intensively used for crop protection.^9,19,20^ In 2019, these were, respectively, the second, fourth, seventh, and tenth most sold triazoles in agriculture in Belgium.^21^ Both epoxiconazole and propiconazole are now banned from use at the EU level under Regulation (EC) No. 1107/2009, because of suspected fertility, developmental, and endocrine perturbation properties.^22^ The largest volumes of azoles are sold in Asia and Europe, accounting for more than two-thirds of all azoles used worldwide.^23^

Since all azoles target the enzyme lanosterol-14-alpha-demethylase (*cyp*51), which inhibits the biosynthesis of ergosterol, cross-resistance is seen between the medical and agricultural azoles.^8,24,25^*Aspergillus fumigatus* has two *cyp*51 isoforms, namely c*yp*51A and *cyp*51B. Most azole-resistant strains display mutations in the *cyp*51A gene, although other resistance mechanisms have been described.^26–30^ C*yp*51A gene mutations can be either tandem repeats in the gene promoter, single-nucleotide polymorphisms, or both.^11^ The most frequently detected tandem repeat mutations in the promotor region are TR34/L98H and TR46/Y121F/T289A.^7,11,24^

Subsequently, multiple articles have described the presence of so-called hotspots for azole resistance in different environments across Europe, namely flower bulb waste, green waste, and woodchips.^7,19,31,32^ Hotspots can be defined as settings that support the growth, the reproduction, and the dispersal of a population of *A. fumigatus*. Furthermore, the conditions must be as such that selection of resistant phenotypes occurs within a mixed population of susceptible and resistant strains, and the substrate must contain residues of demethylation inhibitors (DMIs) at sufficient concentrations.^32^ To date, limited data are available on the presence of azole-resistant *A. fumigatus* in the environment in Belgium.

The use of these azoles in ornamental plants for manipulating the shape, size, and aesthetic quality, in countries such as the Netherlands and Denmark, is significant and is linked to their market value.^23^ Consequently, waste materials from agriculture and horticulture, along with input material from wood, forestry, paper, household, and garden waste, are likely to contain fungicide residues. These waste streams are generally composted by industrial composting facilities, where compost is processed in different stages and the rows are frequently turned to improve oxygenation and to control heat and moisture levels. Research from the Netherlands^19,33^ and the United States^34^ showed that composting material is a hotspot for the development and release of a large number of ARAf spores.

From a One Health perspective, the essential role of azole fungicides to secure the food supply should be balanced with the need to preserve the activity of structurally related azoles in the clinical practice.^35,36^ Therefore, it is needed to identify the drivers behind the resistance development against azole fungicides released into the environment. In this context, the European Centre for Disease Prevention and Control advocates active environmental surveillance in all its member states.^37^ To assess the Belgian situation, a pilot study was carried out in the environment to map the Belgian reservoirs of ARAf. This was addressed by assessing the susceptibility pattern and associated genetic mutations of potential ARAf isolates from air, soil, and/or compost samples from an agricultural field, a horticultural site, and two industrial composting facilities. We describe the need for harmonization and standardization of environmental surveillance methods in Europe and recommend the development of a European surveillance network.

## Materials and methods

### Environmental sampling

Selection of the sampling sites was performed based on a literature search for hotspots of ARAf. Soil, air, and compost were collected from seven different sampling locations comprising two commercial compost processing facilities of green waste and manure (Antwerp region and Flemish Brabant), horticultural (East Flanders), and agricultural crops (Walloon Brabant) (Table 1). Both the horticultural and agricultural sampling campaigns were performed in experimental sites where known concentrations of different azoles and growth inhibitors were applied in the framework of experimental efficacy trials under the Good Agricultural Practice. A blank sample, in the absence of fungicide application, has been included in the horticultural (only hibiscus) and the agricultural sampling campaigns. The level of azoles or plant growth regulators (PGRs) in the compost samples has not been determined in this pilot project but will be included in future projects.

**Table 1.** Environmental sampling locations and sampling approaches.

Environmental sampling locations		Sampling dates	Sampling approach	Air impaction method
Compost	Facility 1	August 2020	Air and compost	RCS^®^
	Facility 2	April 2021	Air and compost	RCS^®^
Agriculture	Wheat	May–July 2022*	Air and soil	MAS-100NT^®^
Horticulture	Roses	June 2020^†^	Air and substrate	MAS-100NT^®^
	Hibiscus	June 2020^‡^	Air and substrate	MAS-100NT^®^
	Primula	January 2021^§^	Air and substrate	MAS-100NT^®^
	Residue heap^¶^	June 2020	Soil	NA

* Four sampling campaigns were performed: before treatment (11 May 2022), 6 days after treatment (18 May 2022), 3 weeks after treatment (2 June 2022), and during harvest (28 July 2022).

† Sampling was performed 3 days after last treatment.

‡ Sampling was performed 28 days after last treatment.

§ Sampling was performed 41 days after last treatment.

¶ The residue heap acts as a bioremediation location where leftovers of plant protection product (PPP) spray dilutions are poured in order to eliminate PPP waste; MAS-100NT® = Microbial Air Sampler; RCS® = Reuter Centrifugal Sampler; NA = not applicable.

#### Compost sampling

The first compost company treated household waste, green waste, and animal manure. The compost was processed in five stadia going from fresh material to mature compost over the course of approximately 2 weeks. Temperature and moist content are monitored to ensure a good composting environment. Compost heaps were turned every few days to ensure enough aeration (Table 2). A total of eight samples from the five different maturing stages of composting were taken (Table 3).

**Table 2.** Moisture measurements in composting facility one from the eight sampling locations.

	Street 1	Street 2	Days of maturity
1	Moisture 45%		0–3
2	Moisture 46%	Moisture 41%	3–7
3		Moisture 36%	7–11
4	Moisture 34%	Moisture 35%	11–14
5	Moisture 36%	Moisture 31%	14–17

Compost heaps were turned every few days to ensure enough oxygenation. Stage 1 shows the fresh material and stage 5 the mature compost.

**Table 3.** Total number of CFU of *Aspergillus fumigatus* per sampling type and per medium in two composting facilities.

	Air*			Compost^†^		
Sampling site	Number of samples	RBCA	MC+T^‡^	Number of samples	MC	MC+T
Facility 1—inside	1	121	16	8^§^	7	0
outside	4	692	8			
Facility 2—inside	3	196	29	6^¶^	0	0
outside	2	1	1			

RBCA: Rose Bengal Chloramphenicol Agar; MC: Malt + chloramphenicol; MC+T : Malt + chloramphenicol + tebuconazole.

* Air sampling was performed using the RCS® sampler.

† The sample was directly seeded on both media.

‡ Single colonies were subcultured onto MC+T medium from the RBCA medium.

§ A total of eight samples were taken from five different maturing stages of the compost.

¶ One sample per type of product was analyzed: starting material (green waste and/or animal manure), compost, crude digestate, solid digestate fraction, liquid digestate fraction, and concentrated liquid digestate fraction.

The second company processed green waste derived from diverse waste streams, as well as from food products deemed unsuitable for human or animal consumption, such as rejected, mould-infested, expired, or improperly packaged items. These waste materials were initially subjected to depackaging within the packaging facility, where they were stored for a short period of time resulting in partial fermentation. While the utilization of azoles remained not documented, it primarily encompassed products from conventional agriculture, wherein the application of fungicides is prevalent, and the presence of residues of active substances and/or their metabolites is likely. A total of six samples were taken: starting material, compost, crude digestate, solid digestate fraction, liquid digestate fraction, and concentrated liquid digestate fraction (Table 3).

#### Horticulture and agriculture soil sampling

Experimental wheat crops were treated with combinations of various fungicides and at different concentrations, by foliar spraying. The trials were part of a single-timepoint fungicide management program. A total of 28 plots with different treatment programs were sampled (Supplementary Table 1). One was a control plot, where no antifungal was applied, and one plot was treated with a non-azole PGR only. All other 26 plots received an azole or a combination of azoles.

Four sampling campaigns were carried out: before treatment (11 May 2022), 6 days after treatment (12 May 2022), 21 days after treatment (2 June 2022) and during harvest (28 July 2022). Approximately 5 gr was collected from the topsoil (0–3 cm),^38^ consisting of a pooled sample from three replicates (sampling locations were 30 cm apart within the same experimental plot). Each experimental plot was 30 cm separated from the next.

Experimental horticultural crops (*Primula*, hibiscus, and roses) were treated with combinations of various fungicides and/or PGRs, and at different concentrations, by foliar spraying or pour-on application. The foliar sprays were part of a multiple timepoint-based disease and growth inhibitor management program (Supplementary Tables 2–4). Approximately 5 gr was collected from the substrate. The residue heap, which acts as a bioremediation location where leftovers of plant protection product (PPP) spray dilutions are poured in order to eliminate PPP waste, was also sampled. Approximately 5 gr of soil was collected from the residue heap.

#### Air sampling in field trials and compost facilities

The Microbial Air Sampler (MAS-100NT^®^) (Merck, Darmstadt, Germany) was used for air sampling on agricultural fields and in horticultural greenhouses, whereas in composting facilities, given the high humidity inside, the Reuter Centrifugal Sampler (RCS^®^) was employed (Table 1). The residue heap was located in a greenhouse where doors and windows were open. One general outdoor air sample was taken that represented the outdoor situation, including the air from the horticultural residue heap.

### Isolation of *A. fumigatus*

Air sampling inside and outside of the composting facilities was performed using the RCS^®^, where a standardized volume of 40 l (24 s at 100 l/min) of air was captured inside and 80 l outside (48 s at 100 l/min). The airborne spores were captured on a Rose Bengal Chloramphenicol Agar (RBCA) medium (Biorad, CA, USA) and incubated at 45°C ± 1°C for 48 h ± 2 h. Isolated colonies were transferred afterward onto malt extract agar supplemented with chloramphenicol (0.5 gr/l, MC) and MC supplemented with 4 mg/l of tebuconazole (MC+T, Sigma-Aldrich, Saint‐Louis, MO, USA) and incubated at 48°C ± 1°C for 48 h ± 2 h. Using the MAS-100NT^®^ in agricultural and horticultural sites, a standardized volume of 1000 l of air was captured (10 min at 100 l/min) and airborne spores were collected on MC and MC+T and incubated at 48°C ± 1°C for 48 h ± 2 h. The different air sampling methods were applied because of the high moisture content in the composting facilities, which is not favorable for the MAS100-NT^®^.

To isolate *A. fumigatus* from soil or compost, 1 gr of material was added to 9 ml of 0.85% NaCl + 0.01% Tween 20 solution. After thorough vortexing, 100 µl of the supernatant was seeded onto MC and MC+T agar. Colonies of *A. fumigatus* were recovered from the MC+T plates of both soil and air samples after 48 h ± 2 h of incubation at 48°C ± 1°C. Single colonies were subcultured in MC tubes and stored at 4°C until further analysis. *Aspergillus fumigatus* colonies were identified based on their microscopic and macroscopic characteristics. Matrix-assisted laser desorption/ionization-time of flight mass spectrometry (MALDI-TOF MS) was used to confirm their identity as *Aspergillus* section *Fumigati* using a Microflex LT MALDI-TOF MS instrument (Bruker Daltonics) with the default settings and as described by Cassagne et al.^39^ Their identification was performed with the software Biotyper 4.1 (Bruker Daltonics) using the MSI 2.0 database. The latter is shared online through a free web application (https://msi.happy-dev.fr).^40^

The selection of colonies isolated from MC+T was considered as representative for any ARAf and was subjected for further analysis.

### Minimal inhibitory concentration analysis and *cyp*51A sequencing

All colonies isolated from MC+T were tested by the broth microdilution method according to the European Committee on Antimicrobial Susceptibility Testing (EUCAST) guidelines (E. Def. 9.4).^41^ Briefly, a cell suspension of 1–5 × 10^6^ CFUs (colony‐forming units) per ml was prepared in 10 ml of saline water (8.5 gr/l NaCl) from a 5-day-old subculture on Sabouraud chloramphenicol agar tube. Subsequently, 1 ml of the cell suspension was added and mixed with 10 ml of RPMI‐1640 medium (Sigma‐Aldrich, Saint‐Louis, MO, USA). A total of 100 µl of this cell suspension was added to each well of a 96-well plate containing 100 µl of serial dilutions of the antifungals and a control. The plates were incubated at 35°C ± 1°C for 48 h. The minimal inhibitory concentration (MIC) of four medical azoles (itraconazole [ITC], voriconazole [VOR], posaconazole [POSA], and isavuconazole [ISA]) was determined on colonies isolated from the MC+T medium. The MIC was determined visually as the lowest concentration of antifungal drugs causing complete inhibition of fungal growth. *Pichia kudriazevii* (IHEM 9560 = ATCC 6258), *Candida parapsilosis* (IHEM 3270 = ATCC 22019), and *A. fumigatus* (IHEM 28944 = ATCC 204305) were used as quality control strains. Azole resistance was defined according to the EUCAST clinical breakpoints (v10.0),^42^ for ITC and VOR with MIC >1 mg/l, POSA with MIC > 0.25 mg/l, and ISA with MIC >2 mg/l.

All colonies displaying phenotypic resistance were subjected to c*yp*51A sequencing. DNA extraction was performed using the ZR Fungal/Bacterial DNA Kit (Zymo Research, Freiburg im Breisgau, Germany) following the manufacturer’s instructions. The *cyp*51A gene and its promotor region were amplified and sequenced using five primer pairs (Supplementary Table 5) and the BigDye-Terminator-v3.1 cycle-sequencing kit (Applied Biosystems, Lithuania). Reaction products were purified using magnetic beads (CleanSeq Agencourt®, Beckman Coulter Life Sciences, CA, USA) according to the manufacturer’s instructions and run on an ABI3500 Genetic Analyzer (Applied Biosystems, Lithuania). The obtained sequences were compared to the reference *cyp*51A sequence of wild-type *A. fumigatus* strain ATCC36607 (GenBank accession number: AF338659.1) using the FunResDb database.^43^

## Results

**Figure 1.**
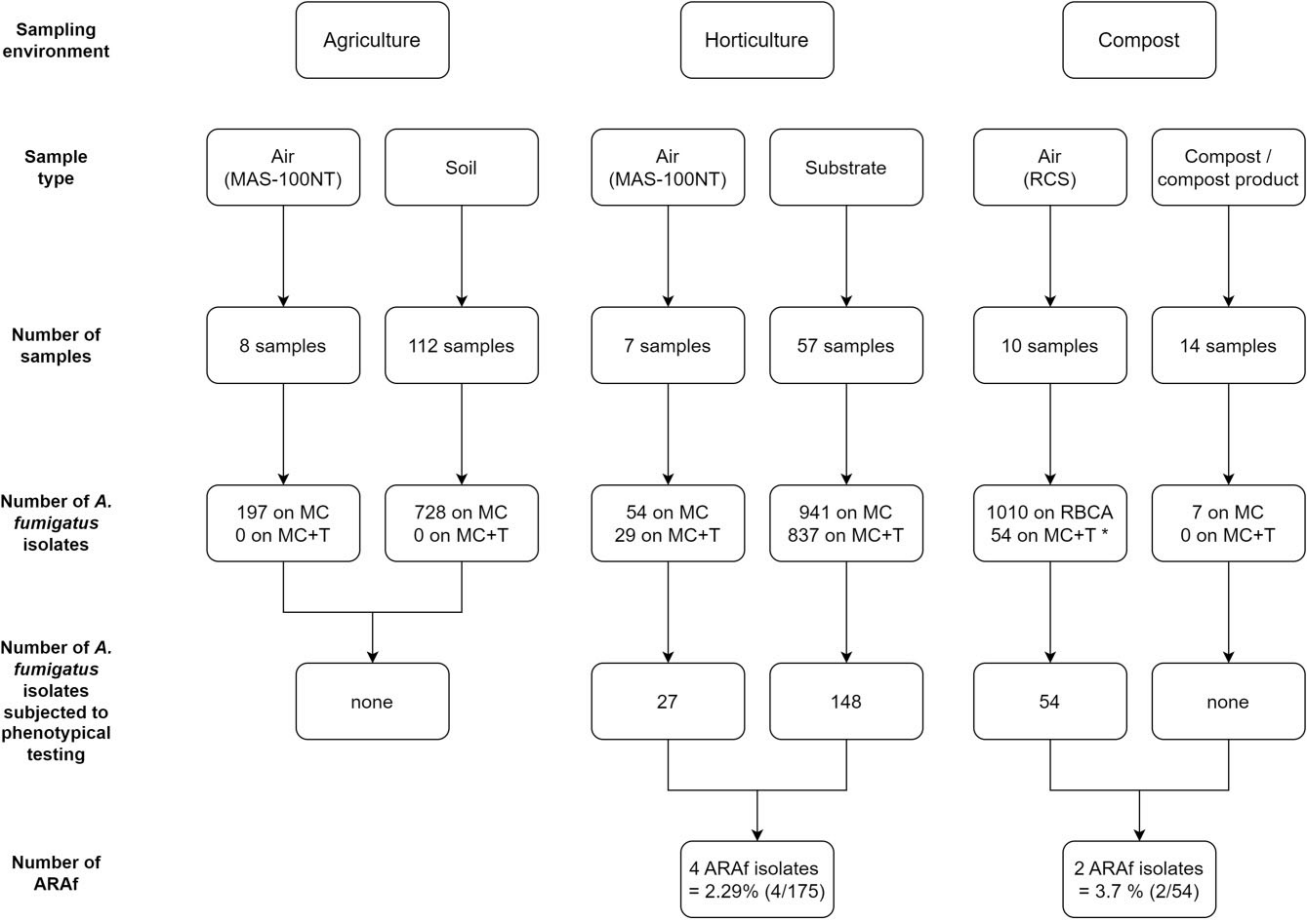
Sampling plan for the detection of (azole-resistant) *Aspergillus fumigatus* in the Belgian environment. MC = malt chloramphenicol; MC+T = malt chloramphenicol + tebuconazole; *A. fumigatus colonies were transferred from the Rose Bengal Chloramphenicol Agar (RBCA) medium to the MC+T medium. Phenotypical resistance testing was performed to determine the MIC value using the EUCAST guidelines (E. Def. 9.4).^41,42^

A total of 232 environmental samples were collected between June 2020 and July 2022 in Belgium: 136 were obtained from agriculture (58.6%), 67 from horticulture (28.9%), and 29 from composting facilities (12.5%) (Fig. 1).

### Compost

*Aspergillus fumigatus* was isolated from two composting facilities in Belgium in both air and compost samples. A high number of airborne spores were captured on the RBCA medium from the first facility (Table 2). However, due to contamination by other fungi, only a subset was analyzed on MC+T medium, resulting in the growth of 24 *A. fumigatus* isolates. A total of 16 colonies were isolated from indoors, whereas 8 were from outside. In addition, a total of eight compost samples were collected from this first composting facility. In the second composting facility, a lower number of colonies (170) were isolated from air samples on the RBCA medium, of which single, non-contaminated colonies were subcultured onto the MC+T medium, resulting in 30 *A. fumigatus* colonies. A total of 29 colonies were isolated from air samples taken indoors. No *A. fumigatus* colonies were detected in the compost samples from both facilities. In total, 54 *A. fumigatus* colonies were subjected to antifungal susceptibility testing. Two isolates displayed phenotypic resistance against at least one medical azole (Table 5).

### Horticulture and agriculture

Air sampling on agricultural and horticultural sampling sites was performed with the MAS-100NT^®^ air sampler, allowing sampling directly on both MC and MC+T from both air and soil or substrate. A total of 29 *A. fumigatus* colonies were isolated from the MC+T medium in air samples from greenhouses in horticulture (Table 4). Regarding the soil/substrate sampling in horticulture, *A. fumigatus* was not recovered from the substrate of roses; nevertheless, *A. fumigatus* was isolated from the substrate of hibiscus plants and in the soil of the residue heap. The highest number of colonies were retrieved from the substrate of *Primula* plants (Table 4). Five *A. fumigatus* colonies were isolated from an air sample and three soil samples from the non-treated hibiscus plants (negative control), however, none were azole-resistant.

**Table 4.** Total number of CFU of *A. fumigatus* per sampling type and per medium in agricultural and horticultural sites.

	Air (CFU/1000 l)			Soil (CFU/10 mg)		
Sampling site	# of samples	MC	MC+T	# of samples	MC	MC+T
Horticulture						
Roses	1	4	5	4	0	0
Hibiscus	4	8	10	14	30	23
*Primula*	2	42	14	18	646	706
Residue heap*	0	NA	NA	6	265	108
Agriculture (wheat)	8	197	0	112	728	0

MC: Malt + chloramphenicol; MC+T : Malt + chloramphenicol + tebuconazole; NA: not applicable;

* The residue heap acts as a bioremediation location where leftovers of plant protection product (PPP) spray dilutions are poured in order to eliminate PPP waste.

A high number of airborne spores (*n* = 197) were captured on the MC medium from agriculture (Table 4). However, no *A. fumigatus* was isolated from the MC+T medium. This was also observed in the soil samples, with a high *A. fumigatus* spore count on the MC medium (*n* = 728), nonetheless, no *A. fumigatus* was isolated from the MC+T medium (Table 4).

### Phenotypic and genotypic resistance testing

Five samples (5/232, 2.16%) contained *A. fumigatus* isolates that showed phenotypical azole resistance against at least one medical azole (Table 5). A total of six isolates that showed phenotypic azole resistance according to the EUCAST clinical breakpoints were detected, resulting in a prevalence of ARAf of 2.62% (6/229 *A. fumigatus* isolates). Sequencing the *cyp*51A gene for those *A. fumigatus* isolates revealed the TR34/L98H mutation in four out of six (66.7%) isolates and one (16.7%) isolate carried the TR46/Y121F/T289A (Table 5). One isolate did not display any known resistance mutations in the *cyp*51A gene. The remaining isolates presented low azole MIC values typical for susceptible isolates.

**Table 5.** Minimal inhibitory concentration results and *cyp*51A mechanism.

			MIC (mg/l)*				
Sampling site	Sample type	Accession nr^†^	VOR	ITC	ISA	POSA	*cyp*51A mutations
Compost (F1)	Air	28 546	8	>16	8	1	TR34/L98H
	Air	28 547	4	>16	8	1	TR34/L98H
Horticulture	Air (hibiscus)	28 954	8	8	2	0,5	TR34/L98H
Horticulture	Soil (residue heap)	28 947	>16	0.5	>64	0,5	TR46/Y121F/T289A
Horticulture	Substrate (*Primula*)	28 553	4	>16	8	1	TR34/L98H
Horticulture	Substrate (*Primula*)	28 554	>16	4	8	2	No mutation detected

MIC = Minimal inhibitory concentration; VOR = voriconazole; ITC = itraconazole; ISA = isavuconazole; POSA = posaconazole; F1 = Facility 1.

* Phenotypical resistance testing was performed to determine the MIC value using the EUCAST guidelines (E. Def. 10.2).^41, 42^

† The isolates were deposited in the BCCM/IHEM collection under the mentioned accession numbers (https://bccm.belspo.be/about-us/bccm-ihem).

## Discussion

Agriculture, horticulture, and composting facilities have been the subject of research on hotspots for azole resistance selection in Europe.^7,19,44,45^ However, the possible presence of ARAf in these environments has not yet been investigated in Belgium. In this pilot study, a total of six isolates, originating from horticulture and composting facilities, displayed phenotypic resistance against at least one medical azole and genetic mutations were present in the *cyp*51A gene. As such, we observed a prevalence of 2.62% (6/229) of ARAf. The isolates were deposited in the BCCM/IHEM collection under the following accession numbers: 28 546, 28 547, 28 553, 28 554, 28 947, and 28 954 (https://bccm.belspo.be/about-us/bccm-ihem). We observed a prevalence of 66.7% (four out of six) of the TR34/L98H mutation and 16.7% (one out of six) of the TR46/Y121F/T289A mutation, which is comparable to what is reported in clinical cases in Belgium, with 83% and 13.87%, respectively.^26^ However, due care must be exercised with these values given the low number of isolates. Burks et al. reported a prevalence of 75% of the TR34/L98H mutation among resistant isolates worldwide and in Europe.^7^ One isolate did not display known resistance mutations in the *cyp*51A gene, which can be an indication of the presence of other resistance mechanisms.^26–30^

The use of fungicides is essential to secure the food supply, as plant pathogenic fungi can cause crop loss of up to 30%.^23,46,47^ However, the development of medical azole resistance has been linked to the use of fungicides, amongst others in agriculture.^9,17^ Different classes of fungicides are used in cropland and in ornamentals, and azole fungicides display a very similar chemical structure and cause cross-resistance with medical azoles. In Germany, Barber et al. estimated the prevalence of ARAf in the environment and in the clinics to be 1.3% and 3.2%, respectively.^44^ The environmental resistance rates reported vary greatly between European countries. Some studies reported almost no resistance, whereas others reported frequencies approaching 14%.^2,48,49^ These differences could be explained by the different nature of the samples. While they are all environmental samples, there is a difference in urban or rural settings or a location with ornamental plants. This is supported by results published by Sewell et al. where rural areas displayed much lower resistance rates (1.1%) as compared to urban areas (13.8%).^48^

In recent years, there has been a growing interest in identifying hotspots for the development of ARAf. Compost of green waste and flower bulb waste have been identified as hotspots.^19^ In this pilot study, two commercial composting facilities were sampled. It was not possible to determine neither the exact nature of the input material of the compost nor the level of azole residues or PGRs in it. Air samples inside the first composting facility did result in many *A. fumigatus* colonies, and two resistant strains carrying the TR34/L98H mutation were detected from the first composting facility. The obtained prevalence of *A. fumigatus* from the composting facilities, generally representing high-load environments, is highly likely to be underestimated in this study; the isolation of *A. fumigatus* was challenged by the presence of other fast-growing species such as *Mucorales* spp., which has been documented before.^50^ These results of the prevalence rates of (azole-resistant) *A. fumigatus* from the composting facilities thus need to be interpreted with caution. The use of the selective flamingo medium for the isolation of *A. fumigatus* could limit the risk of contamination.^51^

Regarding the experimental cropland trials in this study, 128 soil samples and 8 air samples have been collected, resulting in 925 *A. fumigatus* colonies on the MC medium. However, no resistant *A. fumigatus* isolates were found, which is similar to the low prevalence of ARAf isolates described by other researchers in Germany, the United Kingdom, France, and Italy.^20,44,48,52^ The absence of ARAf in the soil of agricultural crops has also been confirmed in root vegetables.^53^ The absence of ARAf in our samples may in part be due to the narrow timeframe of sampling from May to July 2022 covering only spring and the beginning of summer. Other researchers did observe ARAf in samples from cereal soils, but in low concentrations.^54,55^ Fraaije et al. stated that even long-term azole-based foliar fungicide applications did not result in the selection of ARAf strains.^31^

Fungicides, including azoles, are used in horticultural crops to decrease crop losses, but also to increase appearance and longevity.^23^ Most research on ARAf has focused on flower bulb waste from DMI-treated flower bulbs.^19,55^ A number of studies have found that compost made from flower bulb waste treated with DMIs represents a hotspot of ARAf.^19,45^ In the Netherlands, it has been reported that these waste piles produce a high number of spores and a prevalence of up to 24.5% ARAf has been detected in flower bulb waste.^19^ Although we did not study compost heaps of the sampled material that was treated with fungicides and growth inhibitors, we did sample the soil where all residues of the spraying liquid of fungicides were gradually released, thus representing a source of high concentrations of fungicides. Pan-azole-resistant isolates bearing the TR34/L98H mutation were found in this soil. No *A. fumigatus* was detected in the substrate from the roses, which was explained by the different nature of the substrate for the roses, which was a coconut substrate rather than a soil substrate.

We are aware that this pilot study might have some limitations. The most important one is the inconsistency in sampling methods that complicated comparison of prevalence of ARAf in the different environmental settings. This is enforced by the disproportion of sample numbers in agriculture versus horticulture and composting facilities. These limitations highlight the need for clear guidelines on sampling methods in the surveillance of environmental (azole-resistant) *A. fumigatus*. Secondly, this pilot study lacks concentration measurements of azole residues in the soil and compost samples. Future research projects will include more elaborate sampling in horticulture and composting facilities of green waste alongside measurements of azole residues. Additionally, in the present study, we focused on the agricultural and ornamental crops itself, rather than on their waste products. As we found several ARAf originating from ornamental crops, it would be of value to investigate the presence of ARAf in compost heaps originating from ornamental crops.

The World Health Organization’s fungal priority pathogens list ranks *A. fumigatus* as a critical pathogen to guide research.^56^ At EU-level (regulatory PPP approval under Reg. (EC) No 1107/2009), the potential of resistance forming as regards to efficacy is already a data requirement, and the issue of ARAf has recently been extended to the safety assessment of human health.^57^ A more general EFSA mandate to investigate the impact of the use of the azole fungicides, other than as human medicines, on the development of ARAf was formulated accordingly.^58^

To fill existing knowledge gaps in this field, coordinated actions at national and international levels are essential. European standardized guidelines and protocols could contribute to harmonize environmental surveillance, resulting in a better understanding of the epidemiology of *A. fumigatus* resistance. We believe it is necessary to have a clear definition of a hotspot, as well as guidelines on how to measure them. For good management and surveillance, the waste streams within agriculture and horticulture, especially of fungicide-treated crops, must be mapped. These regulations should include a list of all environmental fungicide residues to quantify, including succinate dehydrogenase inhibitors (SDHIs), quinone outside inhibitors (Q_o_Is), and DMIs such as azoles. Furthermore, studies at locations other than the agricultural and horticultural setting should be performed, e.g., sampling of residential gardens and in the hospital environment.^3,59^ Studies on ARAf in retail products are also scarce.^7^

In conclusion, our pilot study reveals that azole-resistant *A. fumigatus* with an environmental background exists in Belgium, highlighting the significance of the One Health perspective to track the development of resistance and prevent its impact in both animal and human health. We report a prevalence of 2.62% of ARAf and the first occurrence of the TR34/L98H and the TR46/Y121F/T289A mutations in the c*yp*51A gene in isolates from horticulture and compost in Belgium. Future work should include more extensive sampling in compost and horticulture with azole residue measurements. Moreover, standardization of environmental surveillance of *A. fumigatus* is lacking and requires urgent developments in collaboration, e.g., with public health authorities. Further work should include the identification and monitoring of the environmental hotspots and their drivers, mapping of the fungicide (not only azoles) use and their waste streams in agricultural and horticultural environments, as well as fungicide residue measurements.

## Supplementary Material

myae055_Supplemental_File
