# Supplementary material for: Detection of pan-azole resistant Aspergillus fumigatus in horticulture and a composting facility in Belgium
Source: Med Mycol. 2024 May 20;62(7):myae055. doi: 10.1093/mmy/myae055 (PMC11223581; doi:10.1093/mmy/myae055)
Supplement: myae055_Supplemental_File [file myae055_supplemental_file.docx]

# Supplementary Material

Supplementary table 1 - All plant protection products, their concentrations and the quantity applied on the experimental wheat cropland

|  |  | Quantity | | | Quantity | | | Quantity | | |
| --- | --- | --- | --- | --- | --- | --- | --- | --- | --- | --- |
| N° | Trade name | Active substance | (g/L) | (g/ha) | Active substance | (g/L) | (g/ha) | Active substance | (g/L) | (g/ha) |
| 1 | Control1 |  |  |  |  |  |  |  |  |  |
| 2 | Eminent | tetraconazole | 125.0 | 125.0 |  |  |  |  |  |  |
| 3 | Proline | prothioconazole | 250.0 | 200.0 |  |  |  |  |  |  |
| 4 | Lenvyor | mefentrifluconazole | 100.0 | 150.0 |  |  |  |  |  |  |
| 5 | Tebucur | tebuconazole | 250.0 | 250.0 |  |  |  |  |  |  |
| 6 | Simveris | metconazole | 90.0 | 90.0 |  |  |  |  |  |  |
| 7 | Narita | difenoconazole | 250.0 | 125.0 |  |  |  |  |  |  |
| 8 | Soleil | tebuconazole | 107.0 | 128.4 | bromuconazole | 167.0 | 200.4 |  |  |  |
| 9 | Kestrel | prothioconazole | 160.0 | 200.0 | tebuconazole | 80.0 | 100.0 |  |  |  |
| 10 | Fandango | prothioconazole | 100.0 | 150.0 | fluoxastrobine | 100.0 | 150.0 |  |  |  |
| 11 | Delaro | prothioconazole | 175.0 | 175.0 | trifloxystrobine | 150.0 | 150.0 |  |  |  |
| 12 | Balaya | mefentrifluconazole | 100.0 | 150.0 | pyraclostrobine | 100.0 | 150.0 |  |  |  |
| 13 | Simveris | metconazole | 90.0 | 90.0 |  |  |  |  |  |  |
|  | Flosul | sulfur | 800.0 | 2400.0 |  |  |  |  |  |  |
| 14 | Simveris | metconazole | 90.0 | 90.0 |  |  |  |  |  |  |
|  | Vertipin | sulfur | 700.0 | 2450.0 |  |  |  |  |  |  |
| 15 | Simveris | metconazole | 90.0 | 90.0 |  |  |  |  |  |  |
|  | Stavento | folpet | 500.0 | 750.0 |  |  |  |  |  |  |
| 16 | Valpura Xpro | bixafen | 125.0 | 125.0 |  |  |  |  |  |  |
| 17 | Revystar Gold | mefentrifluconazole | 100.0 | 150.0 | fluxapyroxad | 50.0 | 75.0 |  |  |  |
| 18 | Revytrex | mefentrifluconazole | 66.7 | 100.1 | fluxapyroxad | 66.7 | 100.1 |  |  |  |
| 19 | Librax | metconazole | 45.0 | 90.0 | fluxapyroxad | 62.5 | 125.0 |  |  |  |
| 20 | Aviator Xpro | prothioconazole | 150.0 | 187.5 | bixafen | 75.0 | 93.8 |  |  |  |
| 21 | Siltra Xpro | prothioconazole | 200.0 | 200.0 | bixafen | 60.0 | 60.0 |  |  |  |
| 22 | Velogy Era | prothioconazole | 150.0 | 150.0 | benzovindiflupyr | 75.0 | 75.0 |  |  |  |
| 23 | Univoq | prothioconazole | 100.0 | 150.0 | fenpicoxamid | 50.0 | 75.0 |  |  |  |
| 24 | Gigant | prothioconazole | 150.0 | 150.0 | isopyrazam | 125.0 | 125.0 |  |  |  |
| 25 | Skyway Xpro | prothioconazole | 100.0 | 125.0 | bixafen | 75.0 | 93.8 | tebuconazole | 100,0 | 125,0 |
| 26 | Priaxor | fluxapyroxad | 75.0 | 75.0 | pyraclostrobine | 150.0 | 150.0 |  |  |  |
|  | Lenvyor | mefentrifluconazole | 100.0 | 100.0 |  |  |  |  |  |  |
| 27 | Variano Xpro | prothioconazole | 100.0 | 175.0 | bixafen | 40.0 | 70.0 | fluoxastrobine | 50,0 | 87,5 |
| 28 | Ascra Xpro | prothioconazole | 130.0 | 195.0 | bixafen | 65.0 | 97.5 | fluopyram | 65,0 | 97,5 |

Supplementary table 2 - All plant protection products and/or plant growth regulators, their concentrations and the quantity applied on hibiscus plants

|  | Trade name | Active substance | Concentration (mL/L) | Method | Quantity (= water volume) | Administration days |
| --- | --- | --- | --- | --- | --- | --- |
| 1 | Vidi parva | 75% seaweed extract | 10.0  10.0 | Pour on  Foliar spray | 100 mL/pot  10 L/are | A, B  C |
| 2 | Tebuphyt | 250 g/l tebuconazole | 8.0 | Foliar spray | 10 L/are | A, B |
| 3 | Fungaflash | 10 g/l imazalil | 20.0 | Foliar spray | 10 L/are | A |
| 4 | Eminent | 125 g/l tetraconazole | 7.5 | Foliar spray | 10 L/are | A, B, C |
| 5 | Geyser | 250 g/l difenoconazole | 5.0 | Foliar spray | 10 L/are | A, B, C |
| 6 | Geyser  Siltac SF | 250 g/l difenoconazole  <75% silicone, siloxanes, polymers | 5.0  1.0 | Foliar spray | 10 L/are | A, B, C |
| 7 | Geyser  Actirob B | 250 g/l difenoconazole  92,8% esterified rapeseed oil | 5.0  1.0 | Foliar spray | 10 L/are | A, B, C |
| 8 | Geyser  Bond | 250 g/l difenoconazole  450 g/l synthetic latex, 100 g/l non-ionic wetting agent | 5.0  1.0 | Foliar spray | 10 L/are | A, B, C |
| 9,10,11,12 | Water | none | none | Foliar spray | 10 L/are | A, B, C |

A = 7/05/2020, B = 15/05/2020 , C = 25/05/2020

Supplementary table 3 - All plant protection products and/or plant growth regulators, their concentrations and the quantity applied on primula plants

|  |  |  | Application days + concentration | | | | | | | | | |
| --- | --- | --- | --- | --- | --- | --- | --- | --- | --- | --- | --- | --- |
|  | Trade name | Active substance | A | B | C | D | E | F | G | H | I | J |
| 1 | Control |  | NA | NA | NA | NA | NA | NA | NA | NA | NA | NA |
| 2 | Bumper 25 EC | 250.0 g/L propiconazole | 0.3 mL | 0.5 mL | 0.5 mL | 0.5 mL | 0.5 mL | 0.5 mL | 0.5 mL | 0.5 mL | NA | 0.5 mL |
| 3 | Alar | 850.0 g/kg daminozide | 3.0 g/L | 3.0 g/L | 3.0 g/L | 3.0 g/L | 3.0 g/L | 3.0 g/L | 3.0 g/L | 3.0 g/L | NA | 3.0 g/L |
|  | Cycocel | 750.0 g/l chlormequat | 1.0 mL | 1.0 mL | 1.0 mL | 1.0 mL | 1.0 mL | 1.0 mL | 1.0 mL | 1.0 mL | NA | 1.0 mL |
| 4 | Caramba | 60.0 g/kg metconazole | NA | 0.5 mL/L | 0.5 mL/L | 0.5 mL/L | 0.5 mL/L | 0.5 mL/L | 0.5 mL/L | 0.5 mL/L | 0.5 mL/L | 0.5 mL/L |
| 5 | Alar | 850.0 g/kg daminozide | 3.0 g/L | 3.0 g/L | 3.0 g/L | 3.0 g/L | 3.0 g/L | 3.0 g/L | NA | NA | NA | NA |
|  | Cycocel | 100.0 g/l chlormequat | 1.0 mL | 1.0 mL | 1.0 mL | 1.0 mL | 1.0 mL | 1.0 mL | NA | NA | NA | NA |
|  | Caryx | 30.0 g/l metconazole + | NA | NA | NA | NA | NA | NA | 1.5 mL | 1.5 mL | NA | 1.5 mL |
|  |  | 210.0 g/l chlormepiquat |  |  |  |  |  |  |  |  |  |  |
| 6 | Fungaflash | 100.0 g/l imazalil | 1.0 mL | 1.0 mL | 1.0 mL | 1.0 mL | 1.0 mL | 1.0 mL | 1.0 mL | 2.0 mL | 2.0 mL | 2.0 mL |
|  | Siltac | polymers | 1.0 mL | 1.0 mL | 1.0 mL | 1.0 mL | 1.0 mL | 1.0 mL | 1.0 mL | 1.0 mL | 1.0 mL | 1.0 mL |
| 7 | Medax top | 300.0 g/l mepiquatchloride + | 1.0 mL | 1.0 mL | 1.0 mL | 1.0 mL | 1.0 mL | 1.0 mL | 1.0 mL | 1.0 mL | 1.0 mL | 1.0 mL |
|  |  | 50.0 g/l prohexadione |  |  |  |  |  |  |  |  |  |  |
| 8 | Tebuphyt | 250.0 g/l tebuconazole | 0.8 mL | 0.8 mL | 0.8 mL | 0.8 mL | 0.8 mL | 0.8 mL | 0.8 mL | 0.8 mL | 0.8 mL | 0.8 mL |
|  | Siltac | polymers | 1.0 mL | 1.0 mL | 1.0 mL | 1.0 mL | 1.0 mL | 1.0 mL | 1.0 mL | 1.0 mL | 1.0 mL | 1.0 mL |
| 9 | Terpal | 305.0 g/l chlormepiquat + | 3.5 mL | 3.5 mL | 3.5 mL | 3.5 mL | 3.5 mL | 3.5 mL | 3.5 mL | NA | NA | NA |
|  |  | 155.0 g/l ethephon |  |  |  |  |  |  |  |  |  |  |

A = 24/09/2020; B= 08/10/2020, C = 14/10/2020; D = 22/10/2020 ; E = 28/10/2020; F = 05/11/2020; G = 13/11/2020; H = 19/11/2020; I = 26/11/2020; J = 04/12/2020; NA = not applicable. All plants were treated using foliar sprays with a concentration of 10 L/are (water volume).

Supplementary table 4 - All plant protection and/or plant growth regulators, their concentrations and the quantity applied on roses

|  | Trade name | Active substance | Concentration (mL/L) | Method | Quantity (= water volume) |
| --- | --- | --- | --- | --- | --- |
| 1 | Fungaflash | 100 g/L Imazalil | 2.0 | Foliar spray | 10 L/are |
| 2 | Closer | 120 g/l sulfoxaflor | 0.2 | Foliar spray | 10 L/are |

Supplementary table 5 - Primer sequences for the c*yp*51A gene and its promotor region of *Aspergillus fumigatus*

| Primer | sequence |
| --- | --- |
| CYP 1F | TCATATGTTGCTCAGCGG |
| CYP 1R | TCTCTGCACGCAAAGAAGAAC |
| CYP 2F | CACTGCAACTCTAATCCTCG |
| CYP 2R | TAACGCAGACTGAGTCAAGC |
| CYP 3F | TTCGGATCGGACGTGGTGT |
| CYP 3R | CGCTGATGGACGAAGACGAA |
| CYP 4F | TGACGGTGACAAGGACTCTC |
| CYP 4R | ACAACCTCGTCGTTCTCCTG |
| CYP 5F | AGTCTTCCTCCGCTCCAGTA |
| CYP 5R | ACACCTATTCCGATCACACC |

F = Forward primer; R = Reverse primer
